# Supplementary material for: Disgust in anorexia nervosa: Testing a theoretical model connecting negative body image to disgust propensity, disgust sensitivity, and self-disgust
Source: PLoS One. 2026 Mar 10;21(3):e0342648. doi: 10.1371/journal.pone.0342648 (PMC12974839; doi:10.1371/journal.pone.0342648)
Supplement: S1 File — (PDF) [file pone.0342648.s001.pdf]

Please note that we could not upload the .spv file to editorial manager, so we decided to add the regression analysis here. We can send the .spv file upon request.

```
Run MATRIX procedure:

*************** PROCESS Procedure for SPSS Version 4.2 beta ***************

          Written by Andrew F. Hayes, Ph.D.       www.afhayes.com
    Documentation available in Hayes (2022). www.guilford.com/p/hayes3

**************************************************************************
Model  : 4
    Y  : BI_2
    X  : DP
    M  : SD

Sample
Size:  126

**************************************************************************
OUTCOME VARIABLE:
 SD

Model Summary
          R       R-sq        MSE          F        df1        df2          p
      .7251      .5258   116.2158   137.5123     1.0000   124.0000      .0000

Model
              coeff         se          t          p       LLCI       ULCI
constant    -7.0816     3.6299    -1.9509      .0533   -14.2663      .1031
DP           1.7345      .1479    11.7266      .0000     1.4417     2.0273

Standardized coefficients
        coeff
DP      .7251

**************************************************************************
OUTCOME VARIABLE:
 BI_2

Model Summary
          R       R-sq        MSE          F        df1        df2          p
      .8262      .6826   182.8336   132.2426     2.0000   123.0000      .0000

Model
              coeff         se          t          p       LLCI       ULCI
constant    -7.5195     4.6223    -1.6268      .1063   -16.6691     1.6301
DP            .4843      .2694     1.7975      .0747     -.0490     1.0176
SD           1.1068      .1126     9.8265      .0000      .8839     1.3298

Standardized coefficients
        coeff
DP      .1326
SD      .7250

************************** TOTAL EFFECT MODEL ****************************
OUTCOME VARIABLE:
 BI_2

Model Summary
          R       R-sq        MSE          F        df1        df2          p
      .6583      .4334   323.7349    94.8374     1.0000   124.0000      .0000

Model
              coeff         se          t          p       LLCI       ULCI
constant   -15.3577     6.0584    -2.5349      .0125   -27.3491    -3.3664
DP           2.4041      .2469     9.7384      .0000     1.9155     2.8927

Standardized coefficients
        coeff
DP      .6583


************** TOTAL, DIRECT, AND INDIRECT EFFECTS OF X ON Y **************

Total effect of X on Y
     Effect         se          t          p       LLCI       ULCI       c_cs
     2.4041      .2469     9.7384      .0000     1.9155     2.8927      .6583

Direct effect of X on Y
     Effect         se          t          p       LLCI       ULCI      c'_cs
      .4843      .2694     1.7975      .0747     -.0490     1.0176      .1326

Indirect effect(s) of X on Y:
       Effect     BootSE   BootLLCI   BootULCI
SD     1.9198      .2354     1.4965     2.4097

Completely standardized indirect effect(s) of X on Y:
       Effect     BootSE   BootLLCI   BootULCI
SD      .5257      .0563      .4218      .6428
```

```
********************** ANALYSIS NOTES AND ERRORS ************************

Level of confidence for all confidence intervals in output:
  95.0000

Number of bootstrap samples for percentile bootstrap confidence intervals:
  5000

------ END MATRIX -----



Run MATRIX procedure:

*************** PROCESS Procedure for SPSS Version 4.2 beta ***************

          Written by Andrew F. Hayes, Ph.D.       www.afhayes.com
    Documentation available in Hayes (2022). www.guilford.com/p/hayes3

**************************************************************************
Model  : 14
    Y  : BI_2
    X  : DP
    M  : SD
    W  : DS

Sample
Size:  126

**************************************************************************
OUTCOME VARIABLE:
 SD

Model Summary
          R       R-sq        MSE          F        df1        df2          p
      .7251      .5258   116.2158   137.5123     1.0000   124.0000      .0000

Model
              coeff         se          t          p       LLCI       ULCI
constant   -41.0498     3.6299   -11.3087      .0000   -48.2345   -33.8652
DP           1.7345      .1479    11.7266      .0000     1.4417     2.0273

**************************************************************************
OUTCOME VARIABLE:
 BI_2

Model Summary
          R       R-sq        MSE          F        df1        df2          p
      .8289      .6870   183.2522    66.4000     4.0000   121.0000      .0000

Model
              coeff         se          t          p       LLCI       ULCI
constant    34.0887     7.2698     4.6890      .0000    19.6961    48.4812
DP            .3424      .3013     1.1363      .2581     -.2541      .9389
SD           1.0743      .1159     9.2716      .0000      .8449     1.3037
DS            .3219      .3089     1.0419      .2995     -.2897      .9335
Int_1        -.0125      .0172     -.7274      .4684     -.0466      .0215

Product terms key:
 Int_1    :        SD       x        DS

Test(s) of highest order unconditional interaction(s):
       R2-chng          F        df1        df2          p
M*W      .0014      .5290     1.0000   121.0000      .4684


****************** DIRECT AND INDIRECT EFFECTS OF X ON Y *****************

Direct effect of X on Y
     Effect         se          t          p       LLCI       ULCI
      .3424      .3013     1.1363      .2581     -.2541      .9389

Conditional indirect effects of X on Y:

INDIRECT EFFECT:
 DP          ->    SD          ->    BI_2

         DS     Effect     BootSE   BootLLCI   BootULCI
    -5.5319     1.9833      .2808     1.4623     2.5524
      .0000     1.8633      .2471     1.3781     2.3497
     5.5319     1.7433      .3018     1.1154     2.2980

      Index of moderated mediation:
        Index     BootSE   BootLLCI   BootULCI
DS     -.0217      .0280     -.0817      .0281

********************** ANALYSIS NOTES AND ERRORS ************************

Level of confidence for all confidence intervals in output:
  95.0000
```

```
Number of bootstrap samples for percentile bootstrap confidence intervals:
  5000

W values in conditional tables are the mean and +/- SD from the mean.

NOTE: The following variables were mean centered prior to analysis:
          DS       SD

NOTE: Standardized coefficients are not available for models with moderators.

------ END MATRIX -----



Run MATRIX procedure:

*************** PROCESS Procedure for SPSS Version 4.2 beta ***************

          Written by Andrew F. Hayes, Ph.D.       www.afhayes.com
    Documentation available in Hayes (2022). www.guilford.com/p/hayes3

**************************************************************************
Model  : 4
    Y  : BI_2
    X  : SD
    M  : BCAQ_AV2

Sample
Size:  126

**************************************************************************
OUTCOME VARIABLE:
 BCAQ_AV2

Model Summary
          R       R-sq        MSE          F        df1        df2          p
      .7605      .5784    28.9293   170.0848     1.0000   124.0000      .0000

Model
              coeff         se          t          p       LLCI       ULCI
constant     9.0069     1.1524     7.8161      .0000     6.7261    11.2877
SD            .4024      .0309    13.0417      .0000      .3413      .4634

Standardized coefficients
        coeff
SD      .7605

**************************************************************************
OUTCOME VARIABLE:
 BI_2

Model Summary
          R       R-sq        MSE          F        df1        df2          p
      .8222      .6760   186.6329   128.2985     2.0000   123.0000      .0000

Model
              coeff         se          t          p       LLCI       ULCI
constant    -2.7159     3.5760     -.7595      .4490    -9.7943     4.3625
SD           1.1790      .1207     9.7697      .0000      .9401     1.4179
BCAQ_AV2      .1855      .2281      .8133      .4176     -.2660      .6370

Standardized coefficients
              coeff
SD             .7722
BCAQ_AV2       .0643

************************** TOTAL EFFECT MODEL ****************************
OUTCOME VARIABLE:
 BI_2

Model Summary
          R       R-sq        MSE          F        df1        df2          p
      .8211      .6742   186.1234   256.6362     1.0000   124.0000      .0000

Model
              coeff         se          t          p       LLCI       ULCI
constant    -1.0451     2.9229     -.3575      .7213    -6.8303     4.7402
SD           1.2537      .0783    16.0199      .0000     1.0988     1.4086

Standardized coefficients
        coeff
SD      .8211


************** TOTAL, DIRECT, AND INDIRECT EFFECTS OF X ON Y **************

Total effect of X on Y
     Effect         se          t          p       LLCI       ULCI       c_cs
     1.2537      .0783    16.0199      .0000     1.0988     1.4086      .8211
```

```
Direct effect of X on Y
     Effect         se          t          p       LLCI       ULCI      c'_cs
     1.1790      .1207     9.7697      .0000      .9401     1.4179      .7722

Indirect effect(s) of X on Y:
             Effect     BootSE   BootLLCI   BootULCI
BCAQ_AV2      .0746      .0876     -.0968      .2482

Completely standardized indirect effect(s) of X on Y:
             Effect     BootSE   BootLLCI   BootULCI
BCAQ_AV2      .0489      .0573     -.0640      .1613

*********************** ANALYSIS NOTES AND ERRORS ************************

Level of confidence for all confidence intervals in output:
  95.0000

Number of bootstrap samples for percentile bootstrap confidence intervals:
  5000

------ END MATRIX -----



Run MATRIX procedure:

*************** PROCESS Procedure for SPSS Version 4.2 beta ***************

          Written by Andrew F. Hayes, Ph.D.       www.afhayes.com
    Documentation available in Hayes (2022). www.guilford.com/p/hayes3

**************************************************************************
Model  : 4
    Y  : BI_2
    X  : SD
    M  : BCAQ_CH

Sample
Size:  126

**************************************************************************
OUTCOME VARIABLE:
 BCAQ_CH

Model Summary
          R       R-sq        MSE          F        df1        df2          p
      .7632      .5825    46.7586   173.0279     1.0000   124.0000      .0000

Model
              coeff         se          t          p       LLCI       ULCI
constant     8.4184     1.4650     5.7462      .0000     5.5187    11.3181
SD            .5160      .0392    13.1540      .0000      .4383      .5936

Standardized coefficients
        coeff
SD      .7632

**************************************************************************
OUTCOME VARIABLE:
 BI_2

Model Summary
          R       R-sq        MSE          F        df1        df2          p
      .8704      .7576   139.6435   192.1649     2.0000   123.0000      .0000

Model
              coeff         se          t          p       LLCI       ULCI
constant    -9.5394     2.8490    -3.3483      .0011   -15.1788    -3.9000
SD            .7331      .1049     6.9874      .0000      .5254      .9407
BCAQ_CH      1.0090      .1552     6.5018      .0000      .7018     1.3162

Standardized coefficients
             coeff
SD           .4801
BCAQ_CH      .4468

************************** TOTAL EFFECT MODEL ****************************
OUTCOME VARIABLE:
 BI_2

Model Summary
          R       R-sq        MSE          F        df1        df2          p
      .8211      .6742   186.1234   256.6362     1.0000   124.0000      .0000

Model
              coeff         se          t          p       LLCI       ULCI
constant    -1.0451     2.9229     -.3575      .7213    -6.8303     4.7402
SD           1.2537      .0783    16.0199      .0000     1.0988     1.4086
```

Standardized coefficients

	coeff
SD	.8211

************** TOTAL, DIRECT, AND INDIRECT EFFECTS OF X ON Y **************

Total effect of X on Y

Effect	se	t	p	LLCI	ULCI	c_cs
1.2537	.0783	16.0199	.0000	1.0988	1.4086	.8211

Direct effect of X on Y

Effect	se	t	p	LLCI	ULCI	c'_cs
.7331	.1049	6.9874	.0000	.5254	.9407	.4801

Indirect effect(s) of X on Y:

	Effect	BootSE	BootLLCI	BootULCI
BCAQ_CH	.5206	.0785	.3712	.6780

Completely standardized indirect effect(s) of X on Y:

	Effect	BootSE	BootLLCI	BootULCI
BCAQ_CH	.3410	.0496	.2465	.4393

********************** ANALYSIS NOTES AND ERRORS ************************

Level of confidence for all confidence intervals in output:
95.0000

Number of bootstrap samples for percentile bootstrap confidence intervals:
5000

------ END MATRIX -----
