# Supplementary material for: Disgust in anorexia nervosa: Testing a theoretical model connecting negative body image to disgust propensity, disgust sensitivity, and self-disgust
Source: PLoS One. 2026 Mar 10;21(3):e0342648. doi: 10.1371/journal.pone.0342648 (PMC12974839; doi:10.1371/journal.pone.0342648)
Supplement: S1 Appendix — Table A. Bivariate relations in the comparison group. Table B. Bivariate correlations in the treatment group. (DOCX) [file pone.0342648.s002.docx]

**S2 Appendix. Bivariate Relations per Group.**

**Table A. Bivariate relations in the comparison group.**

|  | **2.** | **3.** | **4.** | **5.** | **6.** |
| --- | --- | --- | --- | --- | --- |
| 1. Disgust propensity | .67 | .43 | .27 | .31 | .40 |
| 2. Disgust sensitivity |  | .61 | .43 | .45 | .55 |
| 3. Self-disgust |  |  | .58 | .58 | .77 |
| 4. Negative body image |  |  |  | .27 | .68 |
| 5. Body avoidance |  |  |  |  | .41 |
| 6. Body checking |  |  |  |  |  |

**Table B. Bivariate relations in the treatment group.**

|  | **2.** | **3.** | **4.** | **5.** | **6.** |
| --- | --- | --- | --- | --- | --- |
| 1. Disgust propensity | .45 | .52 | .45 | .50 | .54 |
| 2. Disgust sensitivity |  | .20 | .25 | .34 | .20 |
| 3. Self-disgust |  |  | .55 | .55 | .31 |
| 4. Negative body image |  |  |  | .43 | .60 |
| 5. Body avoidance |  |  |  |  | .44 |
| 6. Body checking |  |  |  |  |  |
