# Supplementary material for: Disgust in anorexia nervosa: Testing a theoretical model connecting negative body image to disgust propensity, disgust sensitivity, and self-disgust
Source: PLoS One. 2026 Mar 10;21(3):e0342648. doi: 10.1371/journal.pone.0342648 (PMC12974839; doi:10.1371/journal.pone.0342648)

**S3 Appendix. EDE-Q Global scores per Group.**

**Table A. EDE-Q Global scores per Group.**

|  | M | SD | Range |
| --- | --- | --- | --- |
| Treatment group | 3.91 | 1.02 | 1.50-5.64 |
| Comparison group | 1.33 | 0.13 | 0.00-3.82 |

**Fig A. EDE-Q global score of treatment group.**


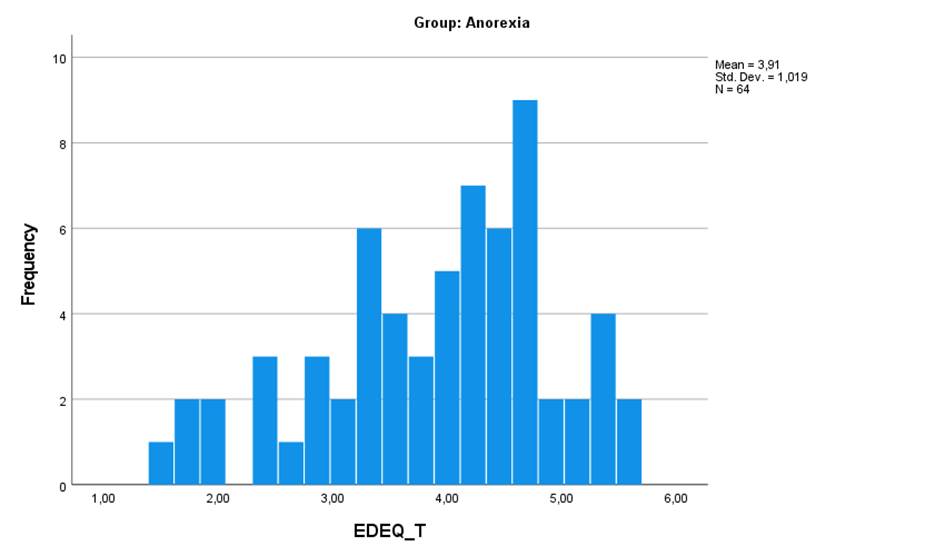


**Fig B. EDE-Q global score of comparison group.**


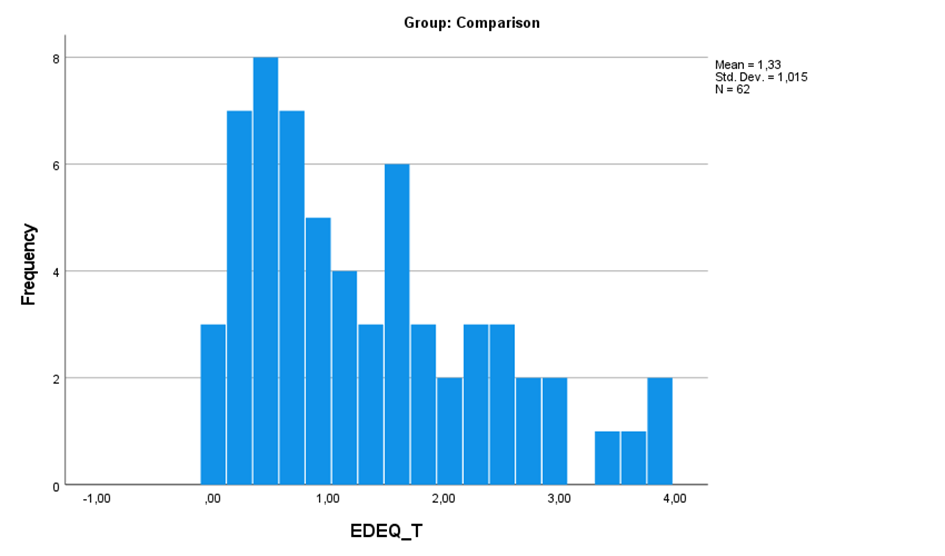

Supplement: S2 Appendix — Table A. EDE-Q global scores per group. Fig A. EDE-Q global score of the treatment group. Fig B. EDE-Q global score of the comparison group. (DOCX) [file pone.0342648.s003.docx]
