# Supplementary material for: Disgust in anorexia nervosa: Testing a theoretical model connecting negative body image to disgust propensity, disgust sensitivity, and self-disgust
Source: PLoS One. 2026 Mar 10;21(3):e0342648. doi: 10.1371/journal.pone.0342648 (PMC12974839; doi:10.1371/journal.pone.0342648)
Supplement: S3 Appendix — Table A. Results of the mediation analysis with group added as covariate. Table B. Results of the moderated mediation analysis with group added as covariate. (DOCX) [file pone.0342648.s004.docx]

**S4 Appendix. Main analyses with group added as covariate**

**Table A. Results of the mediation for the main analysis with group added as covariate.**

| **Path/effect** | **B (SE)** | **t** | **p-value** | **95%CI** |
| --- | --- | --- | --- | --- |
| *c* Total effect (DP on NBI) | 1.08(.26) | 4.18 | <.001 | 0.56 – 1.59 |
| *c’* direct effect (DP on NBI) | 0.32(.26) | 1.24 | .22 | -0.19 – 0.83 |
| *a* (DP on SD) | 0.93(.15) | 5.98 | <.001 | 0.62 – 1.23 |
| *b* (SD on NBI) | 0.82(.13) | 6.12 | <.001 | 0.55 – 1.08 |
|  | **Effect** | **Boot SE** |  | **Boot CI** |
| *ab* indirect effect (DP on NBI through SD) | 0.76 | 0.18 |  | 0.83 – 1.13 |

*Note.* DP = disgust propensity, NBI = negative body image, SD = self-disgust.

**Table B. Results of the moderated mediation for the main analysis with group added as covariate.**

| **Path/effect** | **B (SE)** | **t** | **p-value** | **95%CI** |
| --- | --- | --- | --- | --- |
| *c’* direct effect (DP on NBI) | 0.15(.29) | 0.52 | .60 | -0.43 – 0.73 |
| *b* (SD on NBI) | 0.78(.14) | 5.70 | <.001 | 0.51 – 1.05 |
| *b2* (DS on NBI) | 0.39(.29) | 1.32 | .19 | -0.19 – 0.97 |
| *b3* (SDxDS on NBI) | -0.003(.02) | -0.20 | .84 | -0.04 – 0.03 |
|  | **Effect** | **Boot SE** |  | **Boot CI** |
| Index of moderated mediation | -0.00 | 0.01 |  | -0.04– 0.02 |

*Note.* DP = disgust propensity, NBI = negative body image, DS = disgust sensitivity, SD = self-disgust.
