# Supplementary material for: Disgust in anorexia nervosa: Testing a theoretical model connecting negative body image to disgust propensity, disgust sensitivity, and self-disgust
Source: PLoS One. 2026 Mar 10;21(3):e0342648. doi: 10.1371/journal.pone.0342648 (PMC12974839; doi:10.1371/journal.pone.0342648)
Supplement: S4 Appendix — (DOCX) [file pone.0342648.s005.docx]

**S5 Appendix. Questionnaire items.**

**BCAQ**

1. Ik vermijd plaatsen zoals zwemplassen en stranden.
2. Ik controleer of ik mijn polsen en enkels met een hand kan omsluiten.
3. Ik draag alleen maar donkere, bedekkende kleding of sjaals, om van mijn figuur af te leiden.
4. Ik bevoel/check bepaalde lichaamsdelen zoals mijn buik of heupen.
5. Ik controleer door bepaalde lichaamsbewegingen of mijn vet drilt.
6. Ik toon me niet naakt aan anderen, bijvoorbeeld mijn partner, mijn familie en ook niet bij de dokter of in het ziekenhuis.
7. Ik vraag mijn partner of een vriendin hoe aantrekkelijk hij/zij mij vindt.
8. Ik meet de dikte van mijn dijen met mijn handen of een meetlint.
9. Bij intiem contact met een partner neem ik slechts bepaalde posities in.
10. Ik druk mijn huid tot plooien samen en meet de dikte van de plooien.
11. Ik draag geen kleding die mijn vrouwelijke vormen zichtbaar maakt, zoals spijkerbroeken of strakke tops.
12. Ik vermijd gemeenschappelijke douches, saunabezoeken, zwembaden of thermale baden.
13. Ik trek mijn buik in om te zien hoe het is als mijn buik helemaal plat is.
14. Ik controleer of mijn benen tegen elkaar wrijven als ik loop.
15. Ik draag geen korte kleding voor sport.
16. Ik vergelijk mijn uiterlijk met vrouwen uit tijdschriften of van televisie.
17. Ook in de zomer draag ik kleding die mijn hele lichaam bedekt.
18. Ik word graag gemasseerd en geniet van het lichaamscontact.
19. In de spiegel controleer ik of mijn botten zichtbaar zijn.
20. Ik vraag aan mijn partner of aan vrienden of ik aangekomen ben of weer op een dieet zou moeten gaan.
21. Ik heb alleen maar seks in het donker.
22. Ik vraag aan mijn partner of ik er in bepaalde kleding dik uitzie.
23. Ik vermijd nauw lichaamscontact met andere mensen.
24. Ik controleer of ik cellulitis op mijn dijen heb wanneer ik zit.
25. Ik omsluit mijn bovenarm om de dikte te meten.
26. Bij het winkelen verlaat ik niet met de nieuwe kleding de kleedkamer om mezelf in het openbaar in de spiegel te bekijken.
27. Ik controleer voor de spiegel of mijn dijen elkaar aanraken als ik rechtop sta.

**SDES**

1. Ik vind mijzelf afstotelijk
2. Ik accepteer wie ik ben
3. Ik vind de manier waarop ik me gedraag weerzinwekkend
4. Ik geniet van het gezelschap van anderen
5. Ik accepteer hoe ik eruit zie
6. Delen van mij lichaam zijn vies
7. Ik vind het leuk om buitenhuis te zijn
8. Ik vind de manier waarop ik me gedraag acceptabel
9. Ik wil niet gezien worden
10. Ik ben een sociaal persoon
11. Ik doe vaak dingen die ik weerzinwekkend vind
12. Soms voel ik me blij
13. Ik word er onpasselijk van als ik naar mezelf kijk
14. Soms voel ik me somber
15. Ik haat aspecten van mijn persoonlijkheid
16. Ik denk dat anderen mensen van mij walgen

**EDE-Q**

1. Heb je expres geprobeerd om minder te eten om zo minder te wegen of je lichaamsvormen te veranderen? (Ongeacht of het wel of niet gelukt is)
2. Heb je minstens 8 uren achter elkaar niets gegeten om zo minder te wegen of je lichaamsvormen te veranderen? (De tijd dat je slaapt telt natuurlijk niet mee)
3. Heb je geprobeerd om voedsel dat je lekker vindt niet te eten, om zo minder te wegen of je lichaamsvormen te veranderen?(Ongeacht of het wel of niet gelukt is)
4. Heb je geprobeerd om je aan eetregels te houden om zo minder te wegen of je lichaamsvormen te veranderen? (Ongeacht of het wel of niet gelukt is). Eetregels zijn afspraken die je met jezelf maakt bijvoorbeeld een maximum aan calorieën.
5. Wilde je dat je maag leeg was om zo minder te wegen of je lichaamsvormen te veranderen?
6. Wilde je een absoluut platte buik hebben?
7. Moest je heel veel aan eten of het aantal calorieën denken en kon je daardoor andere dingen niet goed doen? (Bijvoorbeeld je schoolwerk, het doen van een computerspelletje, of lezen)
8. Moest je heel veel aan je figuur en gewicht denken en kon je daardoor andere dingen niet goed doen? (Bijvoorbeeld je schoolwerk, het doen van een computerspelletje, of lezen)
9. Ben je bang geweest de controle over het eten te verliezen? Controle verliezen betekent dat je heel veel moeite had om het eten te laten staan of om te stoppen met eten.
10. Ben je bang geweest om in gewicht aan te komen?
11. Heb je je dik gevoeld?
12. Wilde je gewicht verliezen?
13. Gedurende de afgelopen vier weken (28 dagen), hoe vaak is het voorgekomen dat je het gevoel had zoveel te eten dat anderen dat ook veel zouden vinden?
14. …Tijdens hoeveel van deze keren had je het gevoel dat je de controle over je eten kwijt was?

Controle verliezen betekent dat je heel veel moeite had om het eten te laten staan of om te stoppen met eten.

1. Gedurende de afgelopen vier weken (28 dagen), op hoeveel DAGEN heb je eetbuien gehad?

Een eetbui betekent dat je meer eet dan anderen zouden doen, en dat je tegelijk het gevoel hebt dat je niet meer kan stoppen met eten.

1. Gedurende de afgelopen vier weken (28 dagen), hoe vaak heb je overgegeven om zo minder te wegen of je lichaamsvormen te veranderen?
2. Gedurende de afgelopen vier weken (28 dagen), hoe vaak heb je laxeermiddelen genomen om zo minder te wegen of je lichaamsvormen te veranderen?
3. Gedurende de afgelopen vier weken (28 dagen), hoe vaak heb je veel of hard gesport, met als doel om je gewicht, je lichaamsvormen, of je hoeveelheid vet te veranderen, of calorieën te verbranden?
4. Gedurende de afgelopen vier weken (28 dagen), hoeveel dagen heb je stiekem gegeten?

…. Tel de keren dat je een eetbui had niet mee.

Met stiekem eten wordt bedoeld dat niemand mag weten dat je eet, ook je vrienden of vriendinnen niet.

1. Hoe vaak voelde je je schuldig (het gevoel dat je iets verkeerd gedaan hebt) na het eten?

…. Tel de keren dat je een eetbui had niet mee.

1. Gedurende de afgelopen vier weken (28 dagen), heb je je zorgen gemaakt dat anderen je zagen eten?

…. Tel de keren dat je een eetbui had niet mee.

1. Heeft je gewicht invloed gehad op hoe je over jezelf denkt?
2. Hebben je lichaamsvormen invloed gehad op hoe je over jezelf denkt?
3. Hoe erg zou je het gevonden hebben als je jezelf één keer per week zou moeten wegen (niet minder vaak en niet vaker), gedurende de komende vier weken?
4. Hoe ontevreden heb je je gevoeld over je gewicht?
5. Hoe ontevreden heb je je gevoeld over je lichaamsvormen?
6. Hoe ongemakkelijk heb je je gevoeld als jij je lichaam zag (bijvoorbeeld in de spiegel, in de reflectie van de winkelruit, tijdens het uitkleden, of in bad of onder de douche)?
7. Hoe ongemakkelijk heb je je gevoeld als iemand anders je lichaam zag (bijvoorbeeld in de kleedkamer bij gym, tijdens het zwemmen of bij het dragen van strakke kleding)?
