# Supplementary material for: Disgust in anorexia nervosa: Testing a theoretical model connecting negative body image to disgust propensity, disgust sensitivity, and self-disgust
Source: PLoS One. 2026 Mar 10;21(3):e0342648. doi: 10.1371/journal.pone.0342648 (PMC12974839; doi:10.1371/journal.pone.0342648)
Supplement: Attachment — Submitted filename: Response to Reviewers.docx [file pone.0342648.s007.docx]

Dear Dr. Cruz-Albarran,

On behalf of all authors, I am pleased to submit our revised manuscript PONE-D-24-58634 “Disgust in anorexia nervosa: testing a theoretical model connecting negative body image to disgust propensity, disgust sensitivity, and self-disgust”. Thank you for the positive response and for the opportunity to revise and resubmit.

We appreciate the constructive comments from the reviewers and the editorial team, and have carefully revised the manuscript in light of these suggestions. Detailed responses to each point are provided below.

We look forward to your response.

Yours sincerely,

Fleur Boonstra (Corresponding Author)

| General comments by editor | | Response |
| --- | --- | --- |
| 1 | Please ensure that your manuscript meets PLOS ONE's style requirements, including those for file naming. The PLOS ONE style templates can be found at  https://journals.plos.org/plosone/s/file?id=wjVg/PLOSOne_formatting_sample_main_body.pdf and  https://journals.plos.org/plosone/s/file?id=ba62/PLOSOne_formatting_sample_title_authors_affiliations.pdf | Thank you for your feedback. We carefully revised the manuscript to ensure that it now fully adheres to the PLOS ONE formatting requirements, including the file naming. We appreciate your guidance and hope that the revised version meets the journal’s standards. |
| 2 | We note that the grant information you provided in the ‘Funding Information’ and ‘Financial Disclosure’ sections do not match.  When you resubmit, please ensure that you provide the correct grant numbers for the awards you received for your study in the ‘Funding Information’ section. | Thank you for your comment. We would like to clarify that the authors did not receive any specific funding for this study. We have indicated this in the ‘Financial Disclosure’ before. Please let us know if any additional details are required. |
| 3 | When completing the data availability statement of the submission form, you indicated that you will make your data available on acceptance. We strongly recommend all authors decide on a data sharing plan before acceptance, as the process can be lengthy and hold up publication timelines. Please note that, though access restrictions are acceptable now, your entire data will need to be made freely accessible if your manuscript is accepted for publication. This policy applies to all data except where public deposition would breach compliance with the protocol approved by your research ethics board. If you are unable to adhere to our open data policy, please kindly revise your statement to explain your reasoning and we will seek the editor's input on an exemption. Please be assured that, once you have provided your new statement, the assessment of your exemption will not hold up the peer review process. | In line with your suggestion, we have requested a change to our data repository to ensure that all variables that could potentially identify participants are removed. Once this is completed, the full dataset will be made freely available, and the Data Availability statement will be updated accordingly. |
| 4 | Please include captions for your Supporting Information files at the end of your manuscript, and update any in-text citations to match accordingly. Please see our Supporting Information guidelines for more information: http://journals.plos.org/plosone/s/supporting-information. | Thank you for your comment. We have revised the manuscript to include captions for all Supporting Information files at the end of the document, and updated the in-text citations to match. |
| 5 | If the reviewer comments include a recommendation to cite specific previously published works, please review and evaluate these publications to determine whether they are relevant and should be cited. There is no requirement to cite these works unless the editor has indicated otherwise. | We have carefully reviewed the recommendations by the reviewers and specified whether or not we included this in the manuscript. |

| Reviewer 1 | | Response |
| --- | --- | --- |
| 1 | Definition of disgust sensitivity (line 53–54): The current definition 'the extent to which someone finds the emotion of disgust unpleasant' is somewhat incomplete. It does not fully capture the intensity of an individual’s reaction to disgust or the extent to which they find the experience itself distressing or aversive. Clarifying these aspects would provide a more accurate definition. It is not just about finding disgust "unpleasant" but rather about how much one dreads, avoids, or is emotionally affected by feeling disgusted. | We thank the reviewer for pointing out that the term “unpleasant” does not fully capture the concept of disgust sensitivity. We agree that disgust sensitivity reflects a more intense and specific negative emotional experience. In response to your comment, we have therefore revised the definition to include the extent to which individuals are emotionally affected by experiencing disgust. We also added supporting evidence, including the relationship between disgust sensitivity and anorexia nervosa. We hope you can agree that this revision provides a more accurate and comprehensive description. The change is incorporated in the introduction (lines 52–55), which now reads:  *“Previous research emphasized the importance of distinguishing disgust propensity from disgust sensitivity, which is the extent to which someone negatively evaluates the emotion of disgust or is emotionally affected by feeling disgusted [22]. Increased levels of disgust sensitivity have been observed in individuals with AN compared to non-clinical groups [11,25-28].”* |
| 2 | Link Between Eating Disorders and Anorexia Nervosa (AN): The relationship between eating disorders in general and AN specifically is not clearly established in the introduction. While eating disorders are undoubtedly relevant to the discussion, the connection to AN could be made more explicit and better integrated into your theoretical framework. Specifically, what is the relationship between them. | In line with the reviewer’s suggestion, we clarified in the introduction whether findings refer specifically to anorexia nervosa (AN) or to eating disorders more broadly. These revisions include specifying in line 67 that negative body image is a transdiagnostic factor across eating disorders and indicating in lines 113-115 the diagnoses of the samples in the studies cited. The revised passages now read as follows:  *“Negative body image is suggested to be an essential element of eating disorders in general, as well as a core feature of AN and is thought to be a central factor in its development, maintenance, and relapse (e.g., [35-37]).”*  *“A study including individuals with eating disorders such as anorexia nervosa indicated that these behaviours either repeatedly alternated in individuals with eating disorders or that both behaviours occur simultaneously [50].”* |
| 3 | Bracket Error on Line 65: There is an opening bracket issue: ( instead of [. Please correct this for consistency in your formatting and referencing. | We thank the reviewer for noting this formatting inconsistency. We have corrected the bracket to ensure consistency in formatting and referencing (line 64). |
| 4 | Role of Disgust Sensitivity as a Moderator: When disgust sensitivity’s role in the model is introduced (line 72 - 77) , it is unclear whether it is intended as a moderator in your model. The figure suggests this role, yet it is not explicitly stated in the text. You make it clearer later | In line with the reviewer’s comment, we now explicitly state that following the model disgust sensitivity would act as a moderator. This is incorporated in the introduction (lines 80-83), which now reads as follows:  *“Furthermore, we propose that the relationship between self-disgust and negative body image might be moderated by disgust sensitivity. More specifically, we anticipate that the relationship between self-disgust and negative body image might be most pronounced for individuals with relatively strong disgust sensitivity.”* |
| 5 | Statement on Avoidance Behaviors and Negative Body Image (line 85-88): The statement—'These avoidance behaviours could contribute to the strengthening of a negative body image by preventing: (1) attention toward and appreciation of attractive aspects of one’s body, and (2) habituation to and re-evaluation of ‘aversive’ (disgust eliciting) body parts (cf. [42])'—is not well set up. The preceding discussion on disgust sensitivity does not provide sufficient empirical or theoretical justification for the proposed 'blocking effect' of disgust. To strengthen this argument, it would be beneficial to expand on how disgust sensitivity leads to avoidance behaviors and, in turn, reinforces negative body image. This could be achieved by integrating more relevant literature and clearly describing the underlying mechanisms that link disgust sensitivity, avoidance behaviors, and body image disturbance | We thank the reviewer for this comment and the helpful suggestions. The relationship between disgust sensitivity and avoidance behaviors is primarily based on theoretical reasoning, as empirical studies directly linking disgust sensitivity, avoidance behaviors, and body image disturbances are currently lacking. We have clarified this point in the introduction (lines 83-89) and added a relevant study on disgust sensitivity and avoidance in contamination fear to support the theoretical framework. The revised passage now reads as follows:  *“*Theoretically*, higher levels of disgust sensitivity are thought to motivate people to avoid stimuli that are expected to evoke disgust [22]. This was shown in an experiment including individuals scoring high and low on contamination fear, where disgust sensitivity was predictive of higher fear and behavioural avoidance of contamination-based tasks [42]. Following this line of reasoning, individuals high in disgust sensitivity are expected to show even higher levels of self-disgust induced avoidance of one’s body than individuals scoring low on disgust sensitivity. These avoidance behaviours could contribute to strengthening of a negative body image by preventing: [....].”* |
| 6 | Interpretation of Disgust Sensitivity Findings: The statement—'Although disgust sensitivity was found to be positively related to a negative body image, we did not find evidence for the prediction that disgust sensitivity is a moderator of the relationship between self-disgust and a negative body image.'—raises several important questions that need further clarification: o Does this suggest that disgust sensitivity functions differently in the model than initially expected? If so, how does this align with or contradict prior theoretical assumptions?  o Should the model have been adjusted before conducting this study based on previous findings or pilot data? Given that prior research suggested the model may not perform as expected, what was the rationale for using this approach in the current study? Was it due to limited previous evidence, the need for replication, or differences in the sample population? | Disgust sensitivity indeed appeared to function differently than initially expected based on the theoretical model, and as observed in the previous study. Considering the need for replication, and considering that by including a group of individuals with a clinically diagnosed eating disorder the range in self-disgust, negative body image and disgust sensitivity might be larger, thereby possibly enhancing the sensitivity of the design to detect the hypothesized moderating effect of DS, we chose to retain the original model rather than adjust it on the basis of the absence of a moderating effect in the first empirical test of the model. The double non-finding of a moderation effect of disgust sensitivity on self-disgust and negative body image, now also observed in this mixed (non-)clinical sample, provides valuable information: it offers additional evidence that disgust sensitivity seems not to operate as moderator of the relationship between self-disgust and negative body image, thereby pointing to the relevance of reconsidering the originally proposed role of high DS in the development of a negative body image. We agree with the reviewer that the rationale for retaining the original model as the starting point for the current study could be improved. We therefore revised the relevant section in the introduction (line 101-106), which now reads as follows:  *“Considering the importance of replication studies in psychopathology research, the main aim of the present study was to replicate our prior study and to investigate this theoretical model in a mixed sample of adolescents with and without anorexia nervosa. The inclusion of a mixed (non-)clinical sample was to assure there would be enough variability in our sample. Large variability is recommended when attempting to gain insight into the relationship between continuous variables [45]. The inclusion of a wide range of scores increases power, as well as the generalizability of our results.”* |
| 7 | Rationale for Replicating the Study in a Clinical Sample: The statement: "As a next step, the main aim of the present study was to replicate our prior study and investigate this theoretical model in a clinical sample of adolescents with anorexia nervosa.” While the relevance of this study can be intuited, the rationale for replication in a clinical sample should be more explicitly stated. | We have extended the rationale regarding the inclusion of a clinical sample. The revised introduction now reads as follows (lines 103-106):  *“The inclusion of a mixed (non-)clinical sample was to assure there would be enough variability in our sample. Large variability is recommended when attempting to gain insight into the relationship between continuous variables [45]. The inclusion of a wide range of scores increases power, as well as the generalizability of our results.”* |
| 8 | Justification for Mediation Analysis (Body Checking) The justification for including body checking as a mediator is not well-developed. While body checking behaviors are associated with AN, the paper does not clearly explain why they mediate the relationship between self-disgust and negative body image. The conceptual link between these variables should be explicitly established before introducing the mediation hypothesis. Additionally, if body checking is integral to the theoretical model, a revised figure integrating it should be included. If its inclusion is exploratory, this distinction should be clarified. | The mediation analyses involving body checking and body avoidance were exploratory. We initially tested the original model and subsequently added this line of reasoning as an exploratory extension. This clarification has been added to lines 110-111 which reads as follows:  *“In addition, we conducted exploratory analyses to investigate a possible mediating role of body avoidance and body checking in the relationship between self-disgust and negative body image.”*  We also provided further detail on the potential mediating role of body checking by adding an explanatory sentence (lines 121-124) which reads as follows:  *“The vigilance towards one’s own body in response to self-disgust, may then lower the threshold for experiencing rule-violation thereby promoting the feeling of being (too) fat, which in turn could then directly fuel negative attitudes towards the own body and may thus contribute to the persistence of a negative body image (cf. [15]).”* |
| 9 | Justification for Self-Disgust in the Context of Eating Disorders: The rationale for measuring self-disgust in individuals with eating disorders could be stronger. Is there a specific type of self-disgust that is most relevant to AN? For example, is eating disorder-specific self-disgust more predictive of negative body image than general self-disgust? Providing this clarification would strengthen the theoretical foundation of your study. This might be something to set up earlier in the document that when referring to self-disgust, you are referring to eating disorder related. Or if this measure is more general than the title, could make that clearer. | As argued by Powel et al. (2015), self-disgust may indeed be elicited by various aspects of the self, including one’s dispositional traits, one’s (immoral) behaviours, and one’s physical appearance. Especially, self-disgust as elicited by one’s appearance seemed most relevant for eating disorder problems, as body image concerns are a core problem in ED. In the current introduction this was explained as follows “…*Self-disgust* refers to the appraisal of physical or characterological/behavioural parts of the self as revolting. When individuals experience their own shape and weight as too fat, the confrontation with their own body can elicit intense disgust [28]. In the present study, when mentioning self-disgust, we are specifically referring to such physical/body-related self-disgust.” This was also the reason for using the adjusted SDS that was tailored to assess self-disgust in the context of EDs (the SDES includes reference to visceral qualities and low acceptance of the bodily self (with acceptance used as the opposite of disgust). On the basis of the reviewer’s comment, we further elaborated on this issue in the discussion section. More specifically, in the discussion we acknowledged that self-disgust in AN may not only arise from disgust about one’s appearance but may also be driven by certain dispositional characteristics or (immoral) behaviours. It would be relevant for future research to determine what are the most critical sources for self-disgust in individuals with AN or high levels of body dissatisfaction. This could also shed light on the most promising treatment target(s).  This now reads as follows (lines 437-443):  *“Additionally, it should be acknowledged that we did not differentiate between various aspects of self-disgust. More specifically, self-disgust in AN may not only arise from disgust about one’s appearance but may also be driven by certain dispositional chracteristics and (immoral) behaviours (cf. [29]. We therefore recommend that future research includes more specific measures on different types of self-disgust and negative body image to enable clearer differentiation between constructs. This could also shed light on the most promising treatment target(s).”* |
| 10 | Assumption of Medium-Small Mediation Effect: The study correctly references Fritz & MacKinnon (2007) to justify its statistical power, stating that with the current sample size (N = 126), a medium to medium-small mediation effect could be detected with 80% power. However, I was wondering if the justification could be stronger such as have prior studies in this area found effects of a similar magnitude? | We thank the reviewer for this suggestion, as it enhances transparency by providing a clearer rationale for the statistical power. We have added a justification for the power level estimation in lines 232-234, which now reads as follows:  *“Here, our previous two studies testing the theoretical model also found medium and medium-small sized mediation effects [44].”* |
| 11 | Justification for Mean Centering: The study applies mean centering to manage multicollinearity, but a stronger justification should be included as research has raised concerns about its appropriateness in certain circumstances (see Echambadi & Hess, 2007; Shieh, 2011; Iacobucci et al., 2016). Given these concerns, the study should clearly justify its use of mean centering and whether other techni o Echambadi, R., & Hess, J. D. (2007). Mean-Centering Does Not Alleviate Collinearity Problems in Moderated Multiple Regression Models. Marketing Science, 26(3), 438–445. <https://doi.org/10.1287/mksc.1060.0263> o Shieh, G. (2011). Clarifying the Role of Mean Centring in Multicollinearity of Interaction Effects. British Journal of Mathematical and Statistical Psychology, 64(3), 462–477. <https://doi.org/10.1111/j.2044-8317.2010.02002.x> o Iacobucci, D., Schneider, M. J., Popovich, D. L., & Bakamitsos, G. A. (2016). Mean Centering Helps Alleviate 'Micro' but Not 'Macro' Multicollinearity. Behavior Research Methods, 48, 1308–1317. <https://doi.org/10.3758/s13428-015-0624-x> | We agree with the reviewer's comment regarding mean-centering and its limitations in addressing multicollinearity. In our analysis, mean-centering was applied specifically to facilitate interpretation of the interaction term in the moderated mediation model and to reduce the artificially introduced multicollinearity when interaction terms are included.  Otherwise, there was no multicollinearity present in our data and we applied mean-centering to all variables throughout the analysis for consistency.  We thank the reviewer for pointing this out, as it helped to clarify our reasoning in text. The clarification reads as follows (line 265-267):  *“To facilitate interpretation of the moderated mediation model results and reduce the multicollinearity introduced by its inclusion, variables relevant for this analysis were mean-centered. For consistency, all variables were mean-centered.”* |
| 12 | Comparison to Previous Research: The comparison to prior studies (lines 341–345) is somewhat superficial. A more in-depth discussion linking these findings to existing literature would strengthen this section. Specifically, how do these results align with or challenge previous research on the relationship between self-disgust, body image, and eating disorders? Providing a clearer synthesis of prior findings and positioning this study’s contribution within that context would enhance its impact. | We thank the reviewer for suggesting to provide a more in-depth discussion linking our findings to existing literature. In response to this comment, we have expanded the comparison to prior studies in the discussion (lines 358-68–382), which now reads as follows:  *“In addition to replicating the results of our previous study [44], our findings align with earlier research on the one-to-one associations between self-disgust, disgust propensity, and negative body image. Prior work has shown that individuals with high trait levels of self-disgust, also tend to report elevated disgust propensity [23]. In addition, considering that negative body image is a central feature of AN, the relationship between negative body image and self-disgust may be evident in studies showing that individuals with AN have increased levels of body-directed self-disgust compared to individuals without an eating disorder [28,66] as well as in qualitative findings where individuals with AN describe intense feelings of disgust towards the own body [34]. Furthermore, individuals with a negative body image demonstrated increased levels of body-directed self-disgust both when confronted with their own body [31] and when recalling autobiographical memories related to their body [39-41]. Lastly, there is evidence that negative body image is positively associated with disgust propensity [44]. The current findings add to the existing literature by investigating self-disgust, disgust propensity, and negative body image in one conceptual model**.”* |
| 13 | Implications of the Study: The discussion should more explicitly address the theoretical and practical implications of the findings, particularly regarding disgust sensitivity and the role of body checking and body avoidance in the proposed model. | We agree that especially our non-finding of the moderation of disgust sensitivity has theoretical implications. Therefore, we added a paragraph (lines 389-396) highlighting that future research is needed, which reads as follows:  *“The absence of a moderating effect of disgust sensitivity on the relationship between self-disgust and negative body image in both the current mixed (non-)clinical sample and the previous non-clinical sample [44], suggests that our theoretical model requires adjustment in this particular respect. Previous research does indicate a positive, independent relationship between disgust sensitivity and symptoms of anorexia nervosa such as a negative body image [11, 26,27,28], and therefore it remains relevant to explore if disgust sensitivity should be included somewhere else in the model. Future research is needed to establish whether and how disgust sensitivity contributes to a negative body image.”*    Furthermore, we added that the analysis including body checking was exploratory, and thus replication is needed (line 409-410):  *“As the analysis was exploratory, replication is necessary to determine the robustness of these findings.”*  In addition, we added a theoretical implication of body avoidance (line 423-425):  *“We advocate for the development of self-report instruments that can differentiate between body avoidance in the presence vs. the absence of others so that further research can demonstrate whether this is indeed the case.”* |
| 14 | Relevance of Disgust Sensitivity: Given the non-significant moderation results, should disgust sensitivity still be considered a key variable in this framework? Does its role need to be redefined, or should alternative mechanisms be explored? Clarifying whether disgust sensitivity remains central to the model would provide stronger theoretical coherence. | Disgust sensitivity indeed appeared to function differently than we initially expected. However, given previous research demonstrating positive associations between disgust sensitivity and symptoms of anorexia nervosa (]; Aharoni & Mertz, 2021 [11]; Bektas et al., 2022 [25]; Davey et al., 2009 [26]; Kot et al., 2021 [28; Bell et al., 2017 [27]) as well as our own findings on its link with negative body image (von Spreckelsen et al., 2018 [44]), we believe there remains a valid rationale for keeping disgust sensitivity in the model. That said, we agree with the reviewer that its position within the theoretical framework should be reconsidered.  To address this, we added literature showing a positive independent relationship between disgust sensitivity and symptoms of anorexia nervosa to the introduction (line 54-44), which reads as follows:  “Increased levels of disgust sensitivity have been observed in individuals with AN compared to non-clinical groups [11,25-28].”  In the discussion, we also added a paragraph that highlights the need for future research to gain insight into the role of disgust sensitivity in negative body image. We kindly refer the reviewer back to our response to point 13, where these revisions are detailed. |
| 15 | • Role of Body Checking and Body Avoidance: Are body checking and body avoidance essential components of the model, or do these findings suggest reconsidering their inclusion? If they remain relevant, they should be more clearly integrated into the theoretical framework, either conceptually or through an updated model figure. If their role is more exploratory, this should be explicitly stated. | We thank the reviewer for this comment. The role of body checking and body avoidance in our study is exploratory, and we have clarified this more explicitly in the revised manuscript (lines 396 and 412):  *“A closer, exploratory examination of […].”*  *“Although [.], exploratory analysis revealed that […].”* |
| 16 | • The limitations section would benefit from additional discussion on causality and methodological constraints:  • While an experimental approach could provide stronger evidence, ethical concerns in this context should be acknowledged. Is this actually a doable study? Could be clearer on how this would look | We have added information to the limitations section to clarify the challenges in establishing causality (line 444-445).  *“Therefore, it remains uncertain whether disgust propensity leads to self-disgust and a negative body image, or vice versa.”*  We agree with the reviewer that there are ethical concerns regarding experimentally increasing self-disgust to examine its effect on negative body image. The reverse approach, however, might be feasible: targeting self-disgust to assess whether negative body image decreases. This point has also been added to the paragraph (line 450-452), which reads as follows:  *“**To test this hypothesis, it would be necessary to directly target self-disgust to determine whether a decrease in self-disgust indeed leads to a decrease in negative body image. There are various ways in which body-related self-disgust might be targeted.”* |
| 17 | • Multicollinearity concerns: The presence of high multicollinearity and conceptual overlap between constructs should be discussed in greater detail. How might this have influenced the findings, and what steps could be taken in future research to address these issues? | From a statistical standpoint, we did not find evidence for high multicollinearity in our data. In line with our theoretical framework, correlations between constructs were strong (see Table 3 in text), but multicollinearity was not a concern in our main analysis: VIFs ranged from 1.02 to 2.63.  These values fall below commonly used thresholds for concern, such as 5 or 10 (James et al., 2017).  However, we also recognize that some authors advocate more conservative cutoffs of 2.5, depending on the analysis and context (Johnston et al., 2018). Regarding mediation models, Johnston and colleagues (2018) suggest that collinearity between predictors may be less problematic, as it can reflect meaningful causal pathways rather than statistical redundancy. Hence, less conservative cut-offs are suggested to be more appropriate for mediation models.  In the meantime, it may also be relevant to consider that perhaps the high correlation is (partly) due to the quality of the currently available measures of self-disgust and (negative) body image. We therefore now also explicitly mention in the revision that the current measures might have been suboptimal. We have added this line of reasoning to the discussion, which reads as follows (line 426-443):  *“One of the strengths of the current study is that it is a replication of previous research, thereby adding to the existing literature on (self-)disgust and negative body image. Furthermore, the inclusion of individuals that are in treatment for anorexia nervosa contributed to the generalizability of our results to a clinical population. Alongside its strengths, it is also important to critically evaluate our study. First of all, it should be acknowledged that the measures of self-disgust and negative body image may have been suboptimal for capturing the distinct aspects of these constructs. While we did not find evidence of high multicollinearity in our data, the strong correlations between the constructs (see Table 3) may suggest that there is conceptual overlap in the instruments used. For example, the items of the EDE-Q that were used to assess negative body image (e.g., “How often did you feel fat?”) could be interpreted as assessing (parts of) self-disgust. Vice versa, the Self-Disgust Eating Disorder Scale (SDES) included items that could be interpreted as assessing negative body image (e.g., "I accept how I look") or even broader self-evaluations ("I accept who I am"). Additionally, it should be acknowledged that we did not differentiate between various aspects of self-disgust. More specifically, self-disgust in AN may not only arise from disgust about one’s appearance but may also be driven by certain dispositional traits and (immoral) behaviours (cf. [29]. We therefore recommend that future research includes more specific measures on different types of self-disgust and negative body image to enable clearer differentiation between constructs. This could also shed light on the most promising treatment target(s).”*  *James, G., Witten, D., Hastie, T. J., & Tibshirani, R. J. (2017). An introduction to statistical learning: With applications in R. Springer.*  *Johnston, R., Jones, K., & Manley, D. (2017). Confounding and collinearity in regression analysis: A cautionary tale and an alternative procedure, illustrated by studies of British voting behaviour. Quality &amp; Quantity, 52(4), 1957–1976. https://doi.org/10.1007/s11135-017-0584-6* |
| Reviewer 2 | | Response |
| 1 | In the manuscript “Disgust in anorexia nervosa: testing a theoretical model connecting negative body image to disgust propensity, disgust sensitivity, and self-disgust,” the authors sought to build upon prior work by testing a model of disgust in a sample of girls with anorexia nervosa. Strengths include the matched control group and theoretical basis of the study. The manuscript was well-written and a pleasure to read.  I have two primary concerns. The first is that the study was framed, to a certain extent, on the need to test this model in a clinical sample. These aims were only partially achieved because the sample consisted of half a matched control group.  While the authors do a good job discussing this, it would be helpful to perhaps alter the title and intro to reflect this. I also think it could be helpful to show correlations by group (or maybe even test whether the bivariate correlations differ between groups) to more directly test whether prior findings in healthy populations extend to those with AN. I realize that the authors are underpowered to run their full analyses in the AN subgroup, only. | We adopt a dimensional approach and conceptualize the measured variables as existing on a continuum, with individuals diagnosed with AN typically scoring higher than healthy individuals. Therefore, we assume that results found in healthy individuals differ only in quantity but not quality from those found for individuals diagnosed with AN.  At the same time, we acknowledge that individuals with AN were in treatment at the time of data collection, while healthy individuals were not. This group difference may introduce potential confounding effects. In response to the reviewer’s suggestion, we have therefore compared the bivariate correlations between groups (see Appendix A). While some differences in correlation strength emerged, the overall pattern of associations was consistent across groups, and all key relationships replicated within the treatment group. |
| 2 | My second concern is the discussion of mediation. Mediation isn’t testable in cross-sectional designs (Maxwell & Cole, 2007). However, cross-sectional mediation is statistically the same as testing for a confound/third variable (MacKinnon et al., 2000). I would encourage the authors to avoid mediational language and instead reframe their results as indirect effects or one variable accounting for the relationship of another. | We thank the reviewer for this important point, as we fully agree that the original wording suggested testing mediation, which is not possible with the current design. Accordingly, we have revised the manuscript to use “accounted for” instead of “mediated by” throughout (lines 273, 283, 318-319, 334, 347,353, 355) |
| 3 | Given the varying approaches in the literature, it would be helpful if the authors could indicate how they defined AAN. The EDE doesn’t have a clear algorithm (to my knowledge). | We acknowledge that our original description was unclear and may have suggested that we administered the EDE interview ourselves. We thank the reviewer for pointing this out. In fact, the EDE was conducted at the treatment facility during intake, and we obtained this information for the study. We have clarified this in the methods section and also specified how AAN was defined (lines 142-147), which now reads as follows:  *“Diagnostic information was obtained from the participants’ therapists at Accare, where eating disorder diagnoses are assessed at intake using the child version of the Dutch Eating Disorder Examination (EDE) interview. Atypical AN was diagnosed based on the DSM-5 criteria (i.e., “all of the criteria for anorexia nervosa are met, except that despite significant weight loss, the individual’s weight is within or above the normal range”; [1, p. 353]).”* |
| 4 | In the first paragraph, please note that AN occurs in boys and men as well. The authors acknowledge this in the methods, but I think it’s important to note in the introduction as well. | We agree that there was an inconsistency between the introduction and the methods regarding the inclusion of males and thank the reviewer for noticing. We have added information regarding the prevalence of AN in men at the beginning of the introduction (lines 38-39), which now reads as follows:  “Around 1-4% of women and 0.2-0.7% of men are affected by AN during their lifetime (e.g., [2,3]” |
| 5 | Could the author present EDE-Q Global score Means, SDs, and ranges by group? | We have added the EDE-Q Global score means, standard deviations, and ranges by group to S6 Appendix. |
| 6 | A cut off of 4 seems quite high for the control group, and it seems likely that many of the AN adolescents were below this cut-off based on clinical experience. Could the authors please clarify why they chose a cut off of 4 for the control group? | The cut-off of 4 was based on normative research in the Netherlands, where a global score of 4 has been shown to accurately discriminate individuals with an eating disorder from those without (Aardoom et al., 2012). |
| 7 | Could the authors please clarify which language the questionnaires were administered in? | We have clarified the language of administration in the description of each questionnaire. Specifically, this information has been added for the EDE-Q (line 158), the DPSS-R (line 175), and the SDES (line 186). |
| 8 | Could the authors provide more information about the construct validity of the self-disgust measure used? I had difficulty locating the measure and I’m curious how much it might overlap with self-esteem. The “I accept who I am” sample item does not sound particularly relevant to disgust to me. | Even though the SDES has demonstrated good psychometric qualities (Moncrieff-Boyd et al., 2014), we agree with the reviewer that it is a relatively broad measure. We have now acknowledged this limitation in the discussion (lines 430-442) and emphasized the need for more specific measures of self-disgust in eating disorders, which now reads as follows:  *“First of all, it should be acknowledged that the measures of self-disgust and negative body image have been suboptimal for capturing the distinct aspects of these constructs. While we did not find evidence of high multicollinearity in our data, the strong correlations between the constructs (see Table 2) may suggest that there is conceptual overlap in the instruments used. For example, the items of the EDE-Q that were used to assess negative body image (e.g., “How often did you feel fat?”) could be interpreted as assessing (parts of) self-disgust. Vice versa, the Self-Disgust Eating Disorder Scale (SDES) included items that could be interpreted as assessing negative body image (e.g., "I accept how I look") or even broader self-evaluations ("I accept who I am"). Additionally, it should be acknowledged that we did not differentiate between various aspects of self-disgust. More specifically, self-disgust in AN may not only arise from disgust about one’s appearance but may also be driven by certain psychological traits. We therefore recommend that future research includes more specific measures on different types of self-disgust and negative body image to enable clearer differentiation between constructs. This could also shed light on the most promising treatment target(s).”* |
| Reviewer 3 | | Response |
| 1 | My main concern relates to the authors’ decision to include both individuals with anorexia nervosa and healthy controls in the same mediation model. Given that patients with anorexia nervosa show significantly higher levels across all key variables in the model (disgust propensity, self-disgust, and negative body image), it is possible that group status may act as a confounding variable. In other words, the observed relationships among the variables could be at least partially driven by group differences rather than by genuine associations between psychological constructs. I encourage the authors to provide a clearer justification for combining the two groups within a single model, and—if feasible—to conduct additional analyses that account for this issue. For example, they could run separate mediation models within each group or include group as a covariate or moderator in the model (e.g., using moderated mediation analysis) to test whether the mediation pathway differs between patients and controls. | We agree that it is essential to elaborate on potential group differences and on our decision to not run separate analyses per group.  Our decision to include both individuals with AN and healthy individuals in the analysis was based on a dimensional perspective. We conceptualize the constructs under investigation as continuous variables, with individuals with AN typically scoring higher along these dimensions. Including both groups allowed us to capture a wider range of scores which enhances statistical power (Fisher et al., 2020).  Following the concern that the relationships might be due to group differences, we reran our main analyses with group as a covariate.  The results for the two main analyses with group included as covariate read as follows:  Mediation: A simple mediation analysis served to determine whether the relationship between disgust propensity and negative body image indeed runs via self-disgust (H1). Without self-disgust in the model, results showed that disgust propensity had a statistically significant and positive effect on body image (B = 1.08, SE = 0.26, p < 0.001). When entering self-disgust into the model, the strength of this effect reduced to a statistically non-significant effect (B = 0.32, SE = 0.26, p > .05). Furthermore, results indicated that higher levels of disgust propensity were connected to statistically significantly higher levels of self-disgust (B = 0.93, SE = 0.15, p < 0.001). In turn, higher levels of self-disgust significantly predicted a more negative body image (B = 0.82, SE = 0.13, p < 0.001). The effect of disgust propensity on negative body image via self-disgust was large and statistically significant, as indicated by the 95% bootstrap confidence interval that excluded zero (B = 0.76, SE = 0.18 CI[.44, 1.13]). Together, these results suggest that the effect of disgust propensity on negative body image could be accounted for by self-disgust.  Moderated mediation: The simple mediation model was extended to include disgust sensitivity as a moderator of the relationship between self-disgust and negative body image to test our second hypothesis. Results showed that disgust sensitivity did not statistically significantly interact with self-disgust to predict negative body image (B = -0.003, SE = 0.02, p > .05). Thus, the current results did not support the hypothesis that the relationship between self-disgust and negative body image is moderated by disgust sensitivity.  Although the inclusion of group as a covariate attenuated the effects somewhat, indicating that group differences might explain a small amount of variation in the found effects, the results for models including group as a covariate were consistent with the effects found in the models reported in the text. Also considering power, we therefore report the models not including group as a covariate. We added the additional analysis to S7 Appendix, and mentioned this in a footnote in the main text (page 14):  *“To account for potential effects of group differences, we repeated the main analysis with group as covariate. These results were consistent with the effects found in the models reported in the main text. We therefore included the additional analysis in S7 Appendix.”* |
| 2 | I found the explanation regarding why body avoidance did not emerge as a significant factor in the relationship between self-disgust and negative body image particularly interesting, especially in relation to the nature of the items included in the scale. Would it be possible to conduct an exploratory model focusing only on the items specifically related to self-avoidance? This could provide more insight into whether this specific component plays a role in the mediation pathway. | We thank the reviewer for this suggestion. While we considered this possibility, all items of the BCAQ involve scenarios of body avoidance in the presence of others, as shown in Appendix C where we have listed all items. Therefore, it is not possible to isolate a self-avoidance component for the suggested analysis. We have, however, made this point more explicit in the discussion, where we also advocate for the development of improved measures (lines 423-425), which now reads as follows:  *“We advocate for the development of self-report instruments that can differentiate between body avoidance in the presence vs. the absence of others so that further research can demonstrate whether this is indeed the case.”* |
| 3 | I would encourage the authors to expand the discussion on the potential therapeutic implications of their findings. In particular, I would be interested to know how they envision an intervention specifically targeting self-disgust, and how such an intervention would differ from those aimed at reducing negative body image. | We thank the reviewer for pointing this out, as there are indeed multiple implications to consider, and this was missing from the original version.  We have now added potential therapeutic implications to the discussion (lines 453-464), which read as follows:  *“A first approach to effectively reduce disgust could be via habituation in prolonged exposure therapy [61,62]. Prolonged exposure involves repeated and extended confrontation with the disgust-eliciting stimulus. Considering the inherently disgusting nature of disgust eliciting objects, prolonged exposure is suggested to outperform exposure including expectancy-violation (cf. [15]) (such as used in decreasing fear; e.g. [63]). One way to implement this approach is via body-related mirror exposure, where repeated, therapist-guided confrontation with one’s own body may promote habituation and reduce disgust (cf. [64]). Another promising approach is the use of virtual reality, as it enables repeated and prolonged exposure to representations of one’s body at a higher weight, with the aim of reducing feelings of body-related disgust toward anticipated weight gain. Beyond prolonged exposure, several other strategies have been proposed, either to directly target self-disgust or to more indirectly enhance body appreciation in treatment. For an overview of these treatment suggestions, see [65].”*  Additionally, we acknowledged that targeting self-disgust in isolation is challenging (lines 464-466):  *“However, the effectiveness of these interventions in reducing body-related self-disgust remains to be established, and targeting self-disgust in isolation is inherently challenging within treatment.”* |
| 4 | Page 14, line 293, and page 15, line 309: I believe there may be an error in the references to the figures and tables in relation to the models involving body checking and body avoidance. It seems that the labels may have been accidentally reversed. | We thank the reviewer for noticing this inconsistency, which allowed us to correct the formatting. The figure and table labels for the models involving body checking and body avoidance had indeed been inadvertently reversed. We have corrected these references on page 15 (line 320) and page 16 (line 336). |
