# Supplementary material for: Disgust in anorexia nervosa: Testing a theoretical model connecting negative body image to disgust propensity, disgust sensitivity, and self-disgust
Source: PLoS One. 2026 Mar 10;21(3):e0342648. doi: 10.1371/journal.pone.0342648 (PMC12974839; doi:10.1371/journal.pone.0342648)
Supplement: Attachment — Submitted filename: Response_to_Reviewers_auresp_2.docx [file pone.0342648.s008.docx]

**Response to Reviewer Comments – Revision 2**

**Manuscript Title**: Disgust in anorexia nervosa: testing a theoretical model connecting negative body image to disgust propensity, disgust sensitivity, and self-disgust

**Manuscript ID:** PONE-D-24-58634

**Date:** 11-12-2025

We thank the reviewers for their constructive feedback. Below we provide our point-by-point responses. Additionally, we noticed that the link to our data repository was no longer active. We have corrected this, and the dataset can now be accessed again via the following link: <https://dataverse.nl/previewurl.xhtml?token=2004e7c7-a0aa-46ff-9839-f904bc60db08>.

| **Reviewer 2** | | **Response** |
| --- | --- | --- |
|  | The authors have responded to many of the reviewers’ concerns and the inclusion of sensitivity analyses strengthen the manuscript. A few concerns remain. |  |
| 1 | First, the abstract conclusion and many of the claims in the discussion are not supported by the data- this cross-sectional design cannot support the claim that self-disgust contributes to persistent negative body image in anorexia nervosa. | We appreciate the reviewer’s comment and agree that the cross-sectional design of our study limits the ability to draw conclusions about causal relationships. We have therefore revised the wording in both the abstract and discussion to reflect a more tentative interpretation of our findings and we took care to prevent any suggestion that the current findings would allow causal claims.  In the abstract, we have adjusted the exact phrasing of the conclusion to be more cautious and now state:  Lines 33-34  “Together, these findings are consistent with the view that self-disgust may be an important factor in the persistence of a negative body image in anorexia nervosa.”  Similarly, in the conclusions, we have included further clarification to highlight the potential mechanisms involved, while acknowledging that these findings are correlational.  Lines 470-479  “In conclusion, our findings are consistent with the view that self-disgust may be an important factor in the persistence of a negative body image in anorexia nervosa. Individuals who are prone to experiencing disgust in a variety of situations, seem to also be likely to experience higher self-disgust, and in turn are likely to have a negative body image. In addition, the current pattern of findings is consistent with the view that the relationship between self-disgust and negative body image may run, at least in part, through body checking. Our findings contribute to the understanding of the association between negative body image and self-disgust-related mechanisms. Given the limitations of the current cross-sectional and correlational design, future longitudinal and experimental research is needed to help determine if self-disgust is indeed a central factor in the development and persistence of a negative body image and other eating disorder-related problems.” |
| 2 | Mediational language continues to be used throughout the manuscript- I think this is a significant limitation as there are no prospective or causal pathways in the data. Mediation implies causality. | On the basis of the reviewer’s comment we carefully checked our manuscript for mediational language and corrected the text where relevant to align with the fact that our findings were derived from a cross-sectional study and reflect correlational findings.  Lines 21-22  “Importantly, earlier research in non-clinical samples provided preliminary evidence that this relationship could statistically accounted for by self-disgust.”  Lines 26-28:  “Finally, we explored whether body checking and body avoidance could statistically account for the relationship between self-disgust and negative body image.”  Lines 29-33  “Results showed that (1) the relationship between disgust propensity and negative body image could be statistically accounted for by self-disgust; (2) disgust sensitivity did not moderate the relationship between self-disgust and negative body image, and; (3) the relationship between self-disgust and negative body image could be statistically accounted for by body checking, but not by body avoidance.”  Lines 133-137  “To sum up, we tested whether (1) self-disgust could statistically account for the relationship between disgust propensity and negative body image; (2) disgust sensitivity moderates the relationship between self-disgust and negative body image; (3) body checking could statistically account for the relationship between self-disgust and negative body image; and (4) body avoidance could statistically account for the relationship between self-disgust and negative body image.”  Lines 275-276  “A simple mediation analysis served to determine whether the relationship between disgust propensity and negative body image could be statistically accounted for by self-disgust (H1).”  We also made the following revisions to ensure clarity and avoid the implications of full or partial mediation (as per Hayes, 2017):  Lines 320-322  “In sum, these results indicated that the relationship between self-disgust and negative body image was statistically accounted for by body checking.”  Line 355-357  “Our prior study showed that in undergraduate students with varying levels of body dissatisfaction, the relationship between disgust propensity and negative body image became less strong when self-disgust was added to the model. In the current mixed sample of adolescents with and without AN, the relationship between disgust propensity and negative body image was even no longer significant when self-disgust was added to the model.”  Hayes, A.F. (2017) Introduction to Mediation, Moderation, and Conditional Process Analysis: A Regression-Based Approach. Guilford Press, New York. |
| 3 | I remain concerned about the high threshold for the EDE-Q scores in the control group- the average level of the AN group (3.91) is consistent with the healthy control range (up to 4). Additionally, I think the EDE-Q may not have been scored correctly. Traditionally, it is scored as the average of the four subscales, not an average of all of the items. | We appreciate the reviewer’s concern regarding the EDE-Q scores of the comparison group. However, we would like to emphasize that in our study we followed the recommendations of Fisher et al. (2020) and included data from extreme groups to ensure sufficient variability across the full range of eating pathology and not to directly compare the clinical with the comparison group in a between group analysis (see Fisher et al., 2020). The inclusion of a broad range of scores within both groups increases statistical power and the generalizability of results. In addition, defining the comparison group by a cut-off of 4 on the EDE-Q is recommended by Aardoom et al. (2012). In this way, we followed an asymmetric extreme-group sampling which allowed us to efficiently capture a wide range of symptom severity while analyzing eating pathology as a continuous construct, consistent with a dimensional conceptualization of psychopathology.  That said, after the reviewers’ helpful comment during the previous round of reviews we included an exploratory analysis with group added as a covariate to gain more insight in whether the results of the main analysis could be accounted for at the group level. The analysis showed that the results did not meaningfully differ from the main analysis when group was not added as a covariate. This supports the idea that eating pathology can be measured on a continuum rather than as a dichotomy, and that being in treatment - which is the difference between both groups - did not meaningfully impact the results.  Regarding the scoring of the EDE-Q, we agree with the reviewer that the EDE-Q score is traditionally calculated as the mean of the four subscales. However, we followed the recommendation by Aardoom et al. (2012) to calculate the global EDE-Q score by summing and averaging all individual items. The traditional approach is not theoretically grounded, as factor analyses did not support the theorized four subscales. Either way, the resulting global score appeared to be comparable to the traditional EDE-Q global scores, so both approaches are likely to produce similar results. We chose to sum and average all items because this outweighs the traditional approach in terms of theoretical support.  Fisher JE, Guha A, Heller W, Miller GA. Extreme-groups designs in studies of dimensional phenomena: advantages, caveats, and recommendations. Psychol Methods. 2020;129(1):14-20.  Aardoom JJ, Dingemans AE, Op’t Landt MCS, Van Furth EF. Norms and discriminative validity of the Eating Disorder Examination Questionnaire (EDE-Q). Eat Behav. 2012;13(4):305–9. |
| 4 | Thank you for including Tables A & B in the sensitivity analyses. The correlations between constructs appear to be meaningfully attenuated within group, even to the point that disgust sensitivity and negative body image have a relatively small relationship in the eating disorder group (r = .25). This suggests that the higher correlations in the combined table (e.g., .57) are driven by diagnostic status. As a result, the relationships in the cross-sectional mediation models are driven by diagnostic status. Though under powered, it would be helpful to see what the results look like within just the AN group. This would provide more confidence that the conclusions reached by the authors are not driven by diagnostic group. | Our research question focuses on dimensional relationships among disgust sensitivity, body image, and symptom severity across individuals, rather than on diagnostic group differences. Therefore, we combined groups to maximize variability on these constructs and to test associations across the full continuum of symptom severity. Nevertheless, to ensure the effects were not driven by diagnostic status, we conducted sensitivity analyses controlling for group (and/or within-group analyses) to confirm the robustness of the findings. The pattern of results remained the same between both groups. The results for the two main analyses per group read as follows:  AN group: A simple mediation analysis was conducted to examine whether the association between disgust propensity and negative body image in the AN group could be statistically accounted for by self-disgust (H1 for AN group). Without self-disgust in the model, disgust propensity significantly predicted self-disgust (path a; B = 0.97, SE = 0.20, p < .001). When both disgust propensity and self-disgust were entered as predictors of negative body image, the direct effect of disgust propensity was reduced to a non-significant level (path c’; B = 0.59, SE = 0.32, p = .069). In contrast, self-disgust significantly predicted a more negative body image (path b; B = 0.61, SE = 0.17, p < .001). The indirect effect of disgust propensity on negative body image through self-disgust was statistically significant (path ab; B = 0.59, SE = 0.24, 95% CI [0.19, 1.12]), as the confidence interval did not include zero. Together, these findings suggest that the relationship between disgust propensity and negative body image in the treatment group is statistically accounted for by elevated levels of self-disgust.  Comparison group: A simple mediation analysis was conducted to examine whether the relationship between disgust propensity and negative body image in the comparison group could be statistically accounted for by self-disgust (H1 for comparison group). Results showed that disgust propensity significantly predicted higher levels of self-disgust (path a; B = 0.88, SE = 0.24, p = .0005). When both disgust propensity and self-disgust were entered into the model predicting negative body image, the direct effect of disgust propensity was small and statistically non-significant (path c’; B = 0.10, SE = 0.41, p = .814). In contrast, higher levels of self-disgust significantly predicted a more negative body image (path b; B = 1.00, SE = 0.20, p < .001). The indirect effect of disgust propensity on negative body image through self-disgust was statistically significant, as indicated by the 95% bootstrap confidence interval that excluded zero (path ab; B = 0.87, SE = 0.29, 95% CI [0.33, 1.52]). Together, these findings suggest that the association between disgust propensity and negative body image in the comparison group could be statistically accounted for by elevated levels of self-disgust. |
| 5 | Could the authors please clarify in which mediational pathways they covaried for group? That was not immediately clear from the supplemental tables. | As described in Hayes (2012), the covariate was included in the estimation of all pathways of the mediation model.  Hayes, A. F. (2012). PROCESS: A versatile computational tool for observed variable mediation, moderation, and conditional process modeling [White paper]. Retrieved from http://www.afhayes.com/  public/process2012.pdf |
| 6 | I found lines 376-378 confusing, as I did not think that disgust sensitivity was synonymous with avoidance. | These lines include an interpretation of the finding that disgust sensitivity was not a moderator between self-disgust and negative body image. We made the link with avoidance, since this refers back to our initial reasoning of why disgust sensitivity may contribute to a negative body image that can be found in the introduction (lines 86-97):  “Theoretically, higher levels of disgust sensitivity are thought to motivate people to avoid stimuli that are expected to evoke disgust [22]. This was shown in an experiment including individuals scoring high and low on contamination fear, where disgust sensitivity was predictive of higher fear and behavioral avoidance of contamination-based tasks [42]. Following this line of reasoning, individuals high in disgust sensitivity are expected to show even higher levels of self-disgust induced avoidance of the own body than individuals scoring low on disgust sensitivity. These avoidance behaviors could contribute to the strengthening of a negative body image by preventing: (1) attention toward and appreciation of attractive aspects of the own body, and (2) habituation to and re-evaluation of self-perceived disgusting body parts (cf. [43]). In other words, access to critical information that could counteract pre-existing negative body image beliefs or help develop positive body image beliefs might become blocked. As a result, avoidance behaviors might contribute to the aggravation of a negative body image over time.”  We agree that the absence of a moderating effect of disgust sensitivity between self-disgust and negative body image means that our initial reasoning presented above is not supported by the data.  Therefore, we concluded that our theoretical model requires adjustment in this particular aspect and suggest that future research is needed to establish whether and how disgust sensitivity contributes to a negative body image, in lines 393-400:  “The absence of a moderating effect of disgust sensitivity on the relationship between self-disgust and negative body image in both the current mixed (non-)clinical sample and the previous non-clinical sample [44], suggests that our theoretical model requires adjustment in this particular respect. Previous research does indicate a positive, independent relationship between disgust sensitivity and symptoms of anorexia nervosa such as a negative body image [11,26,27,28], and therefore it remains relevant to explore if disgust sensitivity should be included somewhere else in the model. Future research is needed to establish whether and how disgust sensitivity contributes to a negative body image.” |
